# Influence of *Lactobacillus plantarum* and cellulase on fermentation quality and microbial community in mixed silage of *Solanum rostratum* and alfalfa

**DOI:** 10.3389/frmbi.2024.1510774

**Published:** 2025-01-08

**Authors:** Yuyu Li, Hua Wang, Yandong Zhang, Yu Ji, Lizhu Guo, Lifen Hao, Kejian Lin

**Affiliations:** ^1^ Institute of Grassland Research, Chinese Academy of Agricultural Science, Hohhot, China; ^2^ Key Laboratory of Biohazard Monitoring, Green Prevention and Control for Artificial Grassland, Ministry of Agriculture and Rural Affairs, Hohhot, China

**Keywords:** *Solanum rostratum*, alfalfa, mixed silage, additives, bacterial community

## Abstract

**Introduction:**

Increasing the research on the development and utilization of unconventional feed resources is one of the effective ways for the sustainable development of herbivorous animal husbandry. China is one of the countries most severely impacted by the invasion of the alien plant *Solanum rostratum* Dunal (*S. rostratum*), but this resource has not been used effectively.

**Methods:**

The aim of this study was to investigate the effects of *Lactobacillus plantarum* and cellulase on the fermentation quality and microbial community in mixed silage of *S. rostratum* and alfalfa. Treatments were a control treatment with no additive (CK), *Lactobacillus plantarum* (LP), cellulase (CE), and *Lactobacillus plantarum* in combination with cellulase (L+C), all of which were stored at ambient temperature for 7, 15, 30, and 60 days.

**Results:**

The results showed that the mixture could retain dry matter (DM), crude protein (CP), and water soluble carbohydrates (WSC) content, increase lactic acid (LA) content, decrease pH and alkaloid content, and improve fermentation quality during silage. The use of additives increased the abundance of *Lactobacillus* and *Weissella*, which was related to the improvement of the quality of mixed silage and the degradation of total alkaloids. Differential microbial functions were mainly carbohydrate metabolism, biosynthesis of secondary metabolites and carbon metabolism.

**Conclusion:**

The application of additives and mixed silage provides a new idea for the feed utilization of *S. rostratum*.

## Introduction

Forage is a critical raw material in livestock production, and livestock products are a necessity in people’s daily lives, and a stable supply of forage is of great significance in guaranteeing national food security (Zhao et al., 2024). Against the backdrop of continuing global population growth and food shortages due to climate change, the shortage of feed resources and the irregular supply and high cost of concentrate feeds seriously threaten the sustainable development of the livestock sector (Moselhy et al., 2022). Therefore, scientifically and rationally developing and utilizing unconventional feed resources is one of the effective methods to alleviate the current shortage of conventional feed in China (Sun et al., 2024). This approach can not only effectively address the scarcity of fodder resources but also reduce feeding costs for herbivores, contributing to the advancement of modern livestock farming (Du et al., 2021).

Invasive plants have caused significant damage to the environment and socio-economic conditions in invaded areas, making them one of the five major global environmental issues of the 21st century (Rai and Singh, 2020). Invasive plants often have a stronger competitive ability for ecological resources compared to native plants. Once they invade, they can quickly reproduce and grow due to their inherent advantages, occupying dominant ecological niches and suppressing native plants (Wagg et al., 2014). *S. rostratum* is an annual herbaceous plant in the Solanaceae family and the Solanum genus, and it is recognized as an alien invasive malignant weed (Zhou et al., 2021; Huang et al., 2024). Its reproductive capacity is very high, a mature plant can produce 10,000 to 20,000 seeds in a year, which facilitates population growth and dispersal (Abu-Nassar et al., 2022), and poses significant challenges for management (Rai and Singh, 2020). *S. rostratum* is rich in nutritional value, with a protein content of 15.98% to 17.89%, it also contains various amino acids and vitamins, with vitamin C levels ranging from 0.92 g·kg^-1^~1.42 g·kg^-1^. In autumn and winter, when green forage is scarce, livestock may consume small amounts of *S. rostratum*. However, due to the presence of alkaloids and whole plant covered in sharp spines in *S. rostratum*, it has a very bitter taste and poor palatability, which often leads to livestock avoiding it during the green growth stage (Liu et al., 2021). This characteristic severely limits its utilization as fodder, resulting in significant resource waste and a decrease in its economic value.

At present, it is an effective way to eradicate invasive weeds by using reasonable processing and utilization methods (Raj and Syriac, 2016). Silage not only provides an opportunity to preserve pasture, but also produces lactic acid and other organic acids through the fermentation of microorganisms such as lactic acid bacteria, which can degrade toxic and hazardous substances in pasture, ensure the safety of pasture and improve the palatability of pasture (Huang et al., 2023). A study reported that after 35 days of ensiling Acacia sieberiana, the cyanide content in the raw material decreased from 130.6 mg·kg^-1^to 18.1 mg·kg^-1^. Additionally, ensiling improved the palatability of the forage, resulting in a pleasant aromatic and slightly acidic flavor, as well as a tender and juicy texture (Ngwa et al., 2004). Previous studies reported that the combination of Invasive plant water hyacinth with molasses and pig manure in the ratio of 85:10:5 is the best combination for silage production, after 28 days of silage, it can be used for livestock feeding (Polprasert et al., 1994). A recent experiment by Gottschalk et al. (2018) demonstrated that Silage and additive treatments can be can degrade some pyrrolizidine alkaloids in contaminated grass from eastern groundsel (*Senecio vernalis*). Klevenhusen et al. (2022) found that silage can reduce the content of pyrrolizidine alkaloids in mixed silage feed derived from common ragwort (*Senecio jacobaea*) and eastern groundsel (*Senecio vernalis*). Unconventional feed mulberry leaf silage reduces the anti-nutritional factors in mulberry leaves, improves the palatability of mulberry leaves, reduces nutrient loss and prolongs the storage time (Dong et al., 2020).

Alfalfa (*Medicago sativa* L.), is a prominent legume forage known for its high crude protein content, making it a popular choice for animal feed (Bai et al., 2020). Numerous experiments have shown that to rationally develop and utilize unconventional feeds, mixing unconventional feeds with alfalfa for ensiling not only compensates for the deficiencies between the raw materials and reduces the difficulty of ensiling but also enhances the quality of the silage (Jiang et al., 2024). For example, the mixed silage of alfalfa and maize (*Zea mays*) (Liu et al., 2023), the mixed silage of alfalfa and sunflower straw, and the mixed silage of alfalfa and perennial ryegrass (Fan et al., 2022) have all produced high-quality mixed feed.

During the silage process, various additives have been proposed to ensure the quality of the produced silage feed. *Lactobacillus plantarum* is one of the most widely studied silage additives. It can rapidly initiate the process during the early stages of silage, converting water-soluble carbohydrates (WSC) into lactic acid, which promotes a rapid decrease in pH (Weinberg and Muck, 1996), and it can also inhibit the growth and reproduction of pathogenic bacteria (Yan et al., 2019). Cellulase can degrade the cellulose in the cell walls of forage straw, thereby reducing the fiber content. It also hydrolyzes the polysaccharide materials abundant in the straw into monosaccharides, providing resources for the growth and reproduction of microorganisms and promoting the fermentation process (Khota et al., 2016). The study of Guo et al. (2023) showed that the addition of different starter cultures had significant effects on the fermentation quality and nutrient composition of silage, and might also lead to changes in alkaloid content.

Therefore, this study was performed with the purpose of probing the effect of the combination of *Lactobacillus plantarum* and cellulase on the nutritional quality, fermentation quality, total alkaloid content and microbial community of mixed silage of *S. rostratum* and alfalfa. We hypothesized that compound additives may improve the fermentation quality and nutritional value of silage, promote the degradation of total alkaloid content, and inhibit the growth of other harmful bacteria.

## Materials and methods

### Silage preparation

*S. rostratum* harvested during the flowering period at the at the experimental base of Institute of Grassland Research, Chinese Academy of Agricultural Sciences (40°40’ N, 111°22’ E), Hohhot, Inner Mongolia Autonomous Region, China, on August 14, 2023. Alfalfa (*Medicago sativa* L., Zhongmu No. 1) was planted on June 7, 2020 at the Grassland Institute of the Chinese Academy of Agricultural Sciences in Hohhot (40°58′ N, 111°78′ E). Alfalfa was harvested at the early flowering stage at the third crop on August 15, 2023, with an average of three cuts per year. *S. rostratum* and Alfalfa were harvested with hand sickles. Two forages were harvested and subsequently chopped to a length of 2-3 cm using a hand hay cutter, and then dry them 24-hrs naturally, leading to its moisture content to be reduced to 55-65%. The two forages were thoroughly combined and blended in a 2:3 ratio (wet weight). The treatments were as follows: (1) no additive control (CK); (2) *Lactobacillus plantarum* (LP, Zhongke Jiayi Biological Engineering Co., Ltd., Shandong, China; LP was applied at a level of 10^6^ colony-forming units (cfu) per gram of fresh material (FM)); (3) Cellulase (CE; Yidu Biological Technology Co., Ltd., Hohhot, Inner Mongolia, China; CE activity as 5 × 10^5^ U/g; addition amount: 1 × 10^5^ U/kg fresh material (FM)); (4) *Lactobacillus plantarum*+Cellulase (L+C). All the additives were mixed homogenously with mixed forage. Each treated batch was divided into four replicates (one for backup), which were filled in 1L polythene silage tanks (11 cm diameter × 13.8 cm height) with a filling density of 750 kg/m^3^. Compact and cover with inner and outer lids and seal with tape. A total of 64 tanks (four treatments × four ensiling days × four repeats) were prepared and kept at room temperature (22~25°C). After 7, 15, 30, and 60 days of ensiling, the samples were analyzed.

### Component analysis

The samples underwent a drying process for 72 hours at 65°C to determine their dry matter (DM) content. Crude protein (CP) was analyzed using the Dumas nitrogen determination method with a Dumas-01 model by Gerhardt Analytical Instruments Co., Ltd., Germany. For quantification of acid detergent fiber (ADF), neutral detergent fiber (NDF), and acid detergent lignin (ADL), an ANKOM fiber analyzer (Model: A2000i) from Beijing ANKOM Technology Co., Ltd., China, was employed. Crude fat (EE) analysis utilized an ANKOM fat analyzer (Model: XT15i) from Beijing Anke Borui Technology Co., Ltd. Water-soluble carbohydrates (WSC) were assessed using anthrone colorimetry (Wu et al., 2023). pH levels were measured with a Shanghai Yida Scientific Instruments Co., Ltd. acidity meter (Model: LEICI pH S-3C) in China. Identification of lactic acid (LA), acetic acid (AA), propionic acid (PA), and butyric acid (BA) was conducted using high-performance liquid chromatography with a Waters e2695 model from Massachusetts, USA. Ammonia nitrogen (NH_3_-N) content was determined using the phenol-hypochlorite colorimetric technique (Kozloski et al., 2006). The total alkaloid content in the sample was determined by a kit (Glace Biotechnology Co., LTD., Suzhou). Alkaloids reacted with bromocresol green to form yellow substance, which has a characteristic absorption peak at 415 nm.

### Microbial community analysis

Total DNA extraction, a DNeasy PowerSoil Kit (Qiagen, MD, USA) was used to process according to the manufacturer’s instructions. PCR amplification of bacterial 16S rRNA gene were performed according to Yang et al. (2024) with primers 799F (AACMGGATTAGATACCCKG) and 1193R(ACGTCATCCCCACCTTCC) and PCR conditions were according to the study of (Wang et al., 2024). Purified amplicons were pooled in equimolar amounts and paired-end sequenced on an Illumina Nextseq2000 platform (Illumina, San Diego, USA) according to the standard protocols by Majorbio Bio-Pharm Technology Co. Ltd. (Shanghai, China). The raw sequence data were uploaded to the NCBI archive of sequence reads under study record number PRJNA1170237. The sequencing data were analyzed according to Wang et al. (2019).

### Statistical analysis

In this study, the Quantile-Quantile Plot was used to assess the normality of the data (**Supplementary Figure S1**), the data meet the normal requirements. SPSS 26 was used for two-factor analysis of variance, with 3 replicates per group, and *P* value less than 0.05 was considered significant. The reliability of sample means was evaluated using the standard error of the mean (SEM). Microsoft Excel 2010 was employed for table creation, while graph generation was conducted using Origin 2021 and R 4.1.2. Metabolite identification and annotation were carried out with the Kyoto Encyclopedia of Genes and Genomes (KEGG) compound database, followed by mapping the annotated metabolites to the KEGG pathway database (Nwamba et al., 2021). The correlation between microorganism and quality was analyzed using Spearman correlation coefficient, where * means less than 0.05 and ** means less than 0.01.

## Results

### Characteristics of the fresh materials

The chemical composition and microbial quantity of fresh raw material is shown in **Table 1**. The DM content of *S. rostratum* was 26.97%FW, and the CP content was 16.12%DM, the NDF content was 54.32%DM, the ADF content was 31.54%DM, the WSC content was 6.34%DM, and the TA content was 7.97 g·kg^-1^. The DM content of alfalfa was 34.36%FW, and the CP content was 22.48%DM, the NDF content was 31.57%DM, the ADF content was 28.35%DM, the WSC content was 2.17%DM, and the TA content was 4.64 g·kg^-1^. The DM content of mixtures was 28.63%FW, and the CP content was 20.06%DM, the NDF content was 43.15%DM, the ADF content was 30.58%DM, the WSC content was 3.96%DM, and the TA content was 6.81 g·kg^-1^.

**Table 1:** Chemical and microbial compositions of fresh raw material.

	Items	*S. rostratum*	Alfalfa	SA
Chemical composition	DM (%FW)	26.97	34.36	28.63
	CP (%DM)	16.12	22.48	20.06
	NDF (%DM)	54.32	31.57	43.15
	ADF (%DM)	31.54	28.35	30.58
	WSC (%DM)	6.34	2.17	3.96
	TA(g·kg^-1^ DM)	7.97	4.64	6.81
Microbial counts (lg cfu/g FM)	LAB (lg cfu/g FM)	5.81	5.56	3.87
	A (lg cfu/g FM)	6.37	3.71	5.68
	C (lg cfu/g FM)	6.27	8.39	2.57
	Y (lg cfu/g FM)	5.69	4.59	3.92
	M (lg cfu/g FM)	ND	ND	ND

SA, *S. rostratum* and Alfalfa; FW, fresh weight; DM, dry matter; CP, crude protein; NDF, neutral detergent fiber; ADF, acid detergent fiber; WSC, water-soluble carbohydrates; TA, total alkaloids; LAB, lactic acid bacteria; A, aerobic bacteria; C, coliform bacteria; Y, yeast; M, mold; ND, not detected.

### Effects of additives and silage time on chemical composition of SA silage

The chemical composition of mixed silage is shown in **Table 2**. The additives had significant effects on the contents of DM, CP, ADF, and WSC in mixed silage (*p* < 0.05). The silage time had significant effects on the contents of DM, CP, NDF, ADF, and WSC in mixed silage (*p* < 0.01). The interaction of additives and silage time had significant effects on DM content of mixed silage (*p* < 0.05). The contents of DM, CP, NDF, ADF, and WSC in each group gradually decreased with the extension of silage time, and were significantly lower at 60 days than at 7 days (*p* < 0.05). The contents of DM, CP, NDF, ADF, and WSC in each group gradually decreased with the extension of silage time, and were significantly lower at 60 days than at 7 days (*p* < 0.05). At 7 days of silage, CP content in L+C group was significantly higher than that in control group, WSC content in LP group was significantly higher than that in other groups (*p* < 0.05). At 15 days of silage, WSC content in LP group was significantly higher than that in other groups (*p* < 0.05). At 30 days of silage, the DM content in CE group was significantly higher than that in control group, the ADF content was significantly lower than that in control group (*p* < 0.05), and the WSC content in L+C group was significantly higher than that in control group (*p* < 0.05). At 60 days of silage, the contents of DM, CP and WSC in additive group were significantly higher than those in CK group (*p* < 0.05), while the contents of NDF and ADF were significantly lower than those in CK group (*p* < 0.05).

**Table 2:** Chemical composition of mixed silage.

Items	Treatment	Ensiling days				Significance			
		7	15	30	60	SEM	T	D	T×D
DM (%FW)	CK	26.15Aa	24.50Aab	21.88Cb	21.72Cb	0.210	**	**	*
	LP	24.62Ba	24.11Aab	23.42Bb	23.58Bb				
	CE	25.39ABa	25.28Aa	24.71Aab	24.29ABb				
	L+C	26.03Aa	25.42Aa	24.91Aa	24.74Aa				
CP (%DM)	CK	21.02Ca	20.70Aa	20.49Aa	19.61Bb	0.094	**	**	NS
	LP	21.70ABa	21.01Aab	21.21Aab	20.73Ab				
	CE	21.34BCa	21.35Aa	20.68Ab	20.42Ab				
	L+C	22.09Aa	21.34Aab	21.03Ab	20.98Ab				
NDF (%DM)	CK	40.02Aa	38.39Aab	34.87Abc	31.81Ac	0.713	NS	**	NS
	LP	40.41Aa	38.43Aa	30.10Ab	29.53Bb				
	CE	40.79Aa	36.28Aab	31.61Ab	29.50Bb				
	L+C	41.44Aa	38.21Ab	33.20Ac	29.21Bd				
ADF (%DM)	CK	33.00Aa	31.89Aab	31.25Aab	30.39Ab	0.486	*	**	NS
	LP	31.79Aa	30.40Aa	30.91Aa	26.27Ba				
	CE	34.22Aa	30.82Ab	26.51Bc	26.42Bc				
	L+C	35.09Aa	32.83Ab	27.64Bc	25.45Bd				
WSC (%DM)	CK	3.29Ba	2.89Cb	2.81Bb	2.70Cc	0.051	**	**	NS
	LP	3.88Aa	3.60Ab	3.34ABb	2.86ABc				
	CE	3.37Ba	3.18Bb	3.12ABb	2.90ABc				
	L+C	3.55Ba	3.25Ba	3.48Aa	3.04Ab				

FW, fresh weight; DM, dry matter; CP, crude protein; NDF, neutral detergent fiber; ADF, acid detergent fiber; WSC, water-soluble carbohydrates; CK, no additive control; LP, *Lactobacillus plantarum*; CE, cellulase; L+C, *Lactobacillus plantarum* + cellulase; SEM, standard error of the mean; T, treatments; D, ensiling days; D × T, interaction between treatments and ensiling days; *, significant at 0.05; **, significant at 0.01; means in the same column (A-D) or row (a-d) with different letters differ significantly from each other (*p* < 0.05); NS, not significant.

### Effects of additives and silage time on fermentation composition of SA silage

The fermentation composition of mixed silage is shown in **Table 3**. The contents of pH, LA, AA, PA, and NH_3_-N in mixed silage were significantly affected by additives and silage time (*p* < 0.05). The interaction of additives and silage time had significant effects on the contents of LA and PA in mixed silage (*p* < 0.05). The pH of each group decreased gradually with the extension of silage time, and was significantly lower at 60 days than at 7 days (*p* < 0.05). The contents of LA, AA, PA, and NH_3_-N increased gradually with the extension of silage time, and were significantly higher at 60 days than at 7 days (*p* < 0.05). At 7 days of silage, the pH of LP group was significantly lower than that of control group (*p* < 0.05). At 15 days of silage, the pH of additive group was significantly lower than that of control group (*p* < 0.05). At 30 and 60 days of silage, pH in the LP group was significantly lower than in the other groups (*p* < 0.05). During silage, the content of LA in L+C group was significantly higher than that in control group (*p* < 0.05), while the content of AA, PA, and NH_3_-N in additive group was significantly lower than that in control group (*p* < 0.05).

**Table 3:** Fermentation composition of mixed silage.

Items	Treatment	Ensiling days				Significance			
		7	15	30	60	SEM	T	D	T*D
pH	CK	5.07Aa	4.93Aab	4.72Abc	4.61Ac	0.037	**	**	NS
	LP	4.49Ca	4.39Bb	4.26Cc	4.24Cc				
	CE	4.86ABa	4.60Bb	4.37Bc	4.34Bc				
	L+C	4.71BCa	4.53Bb	4.41Bc	4.28Cd				
LA (%DM)	CK	1.66Cb	2.07Cb	2.28Bb	3.73Ca	0.169	**	**	**
	LP	2.06ABc	2.50ABbc	3.03Bb	4.67Ba				
	CE	1.78BCc	2.14BCbc	2.49Bb	3.73Ca				
	L+C	2.20Ac	2.74Ac	3.92Ab	5.88Aa				
AA (%DM)	CK	0.99Ac	1.21Abc	1.39Aab	1.55Aa	0.041	**	**	NS
	LP	0.59Bc	0.76Cbc	0.85Cb	1.09BCa				
	CE	0.78ABc	0.98Bb	1.15Bab	1.2Ba				
	L+C	0.59Bc	0.71Cbc	0.85cCab	1.03Ca				
PA (%DM)	CK	0.06Ad	0.12ABc	0.19Ab	0.34Aa	0.013	**	**	**
	LP	0.03Ac	0.10ABb	0.12Bb	0.25Ba				
	CE	0.06Ac	0.13Ab	0.17Ab	0.25Ba				
	L+C	0.03Ac	0.08Bbc	0.12Bab	0.14Ca				
NH_3_-N (%TN)	CK	0.44Ad	0.64Ac	0.79Ab	0.85Aa	0.031	**	**	NS
	LP	0.34ABc	0.49Bb	0.50Bb	0.75Ba				
	CE	0.21BCc	0.41BCb	0.55Bb	0.74Ba				
	L+C	0.15Cd	0.29Cc	0.46Bb	0.63Ca				

LA, lactic acid; AA, acetic acid; PA, propionic acid; NH_3_-N, ammonia nitrogen; CK, no additive control; LP, *Lactobacillus plantarum*; CE, cellulase; L+C, *Lactobacillus plantarum* + cellulase; SEM, standard error of the mean; T, treatments; D, ensiling days; D × T, interaction between treatments and ensiling days; *, significant at 0.05; **, significant at 0.01; means in the same column (A-D) or row (a-d) with different letters differ significantly from each other (*p* < 0.05); NS, not significant.

### Effects of additives and silage time on total alkaloid of SA silage

**Figure 1:**
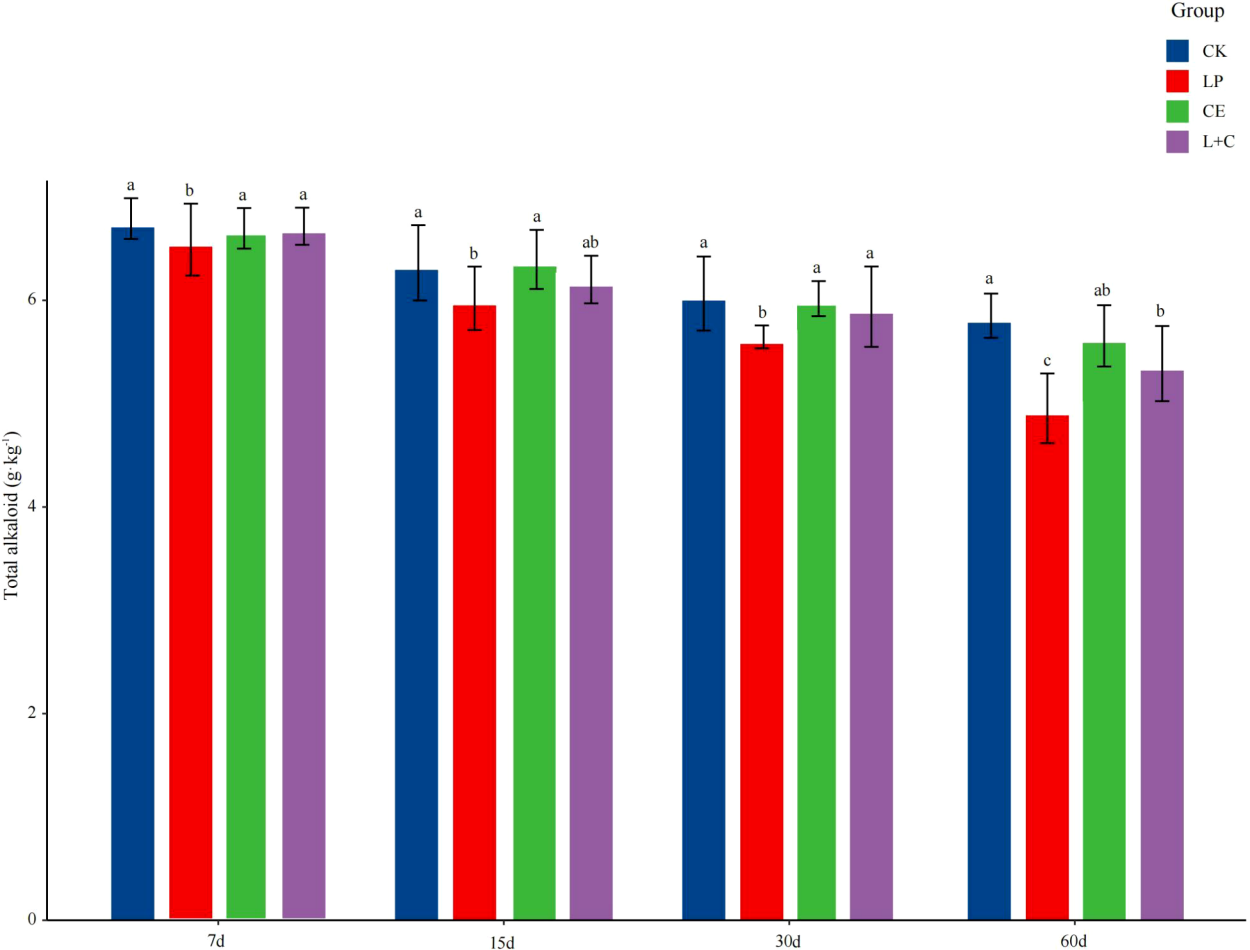
Total alkaloid content of mixed silage. CK, no additive control; LP, *Lactobacillus plantarum*; CE, cellulase; L+C, *Lactobacillus plantarum* + cellulase. Different letters indicated significant difference between groups (*p*< 0.05).

The difference of total alkaloid content in mixed silage is shown in **Figure 1**. With the delay of silage time, the TA content of silage decreased gradually. At 7 days of silage, TA content in LP group was significantly lower than that in other groups, and the content in CK group was the highest (*p* < 0.05). At 15 days of silage, the TA content in LP group was significantly lower than that in CK and CE groups was higher (*p* < 0.05). At 30 days of silage, TA content in LP group was significantly lower than that in other groups (*p* < 0.05), and there was no significant difference in other groups (*p* > 0.05). At 60 days of silage, the TA content in CK group was significantly higher than that in other groups, and the TA content in LP group was the lowest (*p* < 0.05).

### Effects of additives and silage time on microbial community of SA silage

The difference of alpha diversity in mixed silage is shown in **Table 4**. The Sobs, Chao, Shannon, Simpson, and ACE in mixed silage were significantly affected by additives (*p* < 0.05). The interaction between additives and silage time had no significant effect on the alpha diversity of mixed silage (*p* > 0.05). During silage, Sobs, Chao, Shannon and ACE in LP group were significantly lower than those in other groups (*p* < 0.05), and Simpson was significantly higher than those in other groups (*p* < 0.05). Sobs, Chao, Shannon and ACE were significantly higher in the CE group than in the other groups (*p* < 0.05). Sobs, Chao, Shannon and ACE in group CK and group L+C were higher than those in LP group. There was no significant difference in Coverage among all groups (*p* > 0.05).

**Table 4:** Bacterial α diversity of mixed silage.

Items	Treatment	Ensiling days				Significance			
		7	15	30	60	SEM	T	D	T*D
Sobs	CK	52.67Ba	58.33Ba	59.33Ba	66.67Aa	2.131	**	NS	NS
	LP	41.33Ca	46.33Ca	44.33Ca	48.33Ba				
	CE	59.33Aa	65.67Aa	80.67Aa	70.67Aa				
	L+C	61.33Aa	66.33Aa	61.00Ba	47.00Ba				
Chao	CK	52.83Ba	59.10Ba	60.08Ba	67.94Aa	2.119	**	NS	NS
	LP	41.84Ca	47.77Ca	45.05Ca	49.08Ba				
	CE	59.38Aa	66.03Aa	82.47Aa	71.5Aa				
	L+C	61.49Aa	66.63Aa	61.08Ba	48.44Ba				
Shannon	CK	2.1ABa	2.09Aa	1.87Aa	1.88Aa	0.099	**	NS	NS
	LP	0.73Ba	0.57Ba	0.48Ba	0.71Ba				
	CE	2.13Aa	2.16Aa	2.2Aa	2.06Aa				
	L+C	1.70Aab	1.39Aab	1.85Aa	1.21Ab				
Simpson	CK	0.19Ba	0.27Ba	0.34Ba	0.34Ba	0.036	**	NS	NS
	LP	0.75Aa	0.82Aa	0.83Aa	0.77Aa				
	CE	0.22Ba	0.23Ba	0.24Ba	0.3Ba				
	L+C	0.33Ba	0.46Ba	0.33Ba	0.53Ba				
ACE	CK	53.47Ba	59.64Ba	60.73Ba	67.94Ba	2.161	**	NS	NS
	LP	42.77Ca	49.08Ca	45.82Ca	50.51Ca				
	CE	59.69Aa	66.7Aa	83.31Aa	72.69Aa				
	L+C	62.11Aa	67.55Aa	61.55Ba	49.98Ca				
Coverage	CK	0.99	0.99	0.99	0.99	NS	NS	NS	NS
	LP	0.99	0.99	0.99	0.99				
	CE	0.99	0.99	0.99	0.99				
	L+C	0.99	0.99	0.99	0.99				

CK, no additive control; LP, *Lactobacillus plantarum*; CE, cellulase; L+C, *Lactobacillus plantarum* + cellulase; SEM, standard error of the mean; T, treatments; D, ensiling days; D × T, interaction between treatments and ensiling days; *, significant at 0.05; **, significant at 0.01; means in the same column (A-D) or row (a-d) with different letters differ significantly from each other (*p* < 0.05); NS, not significant.

**Figure 2:**
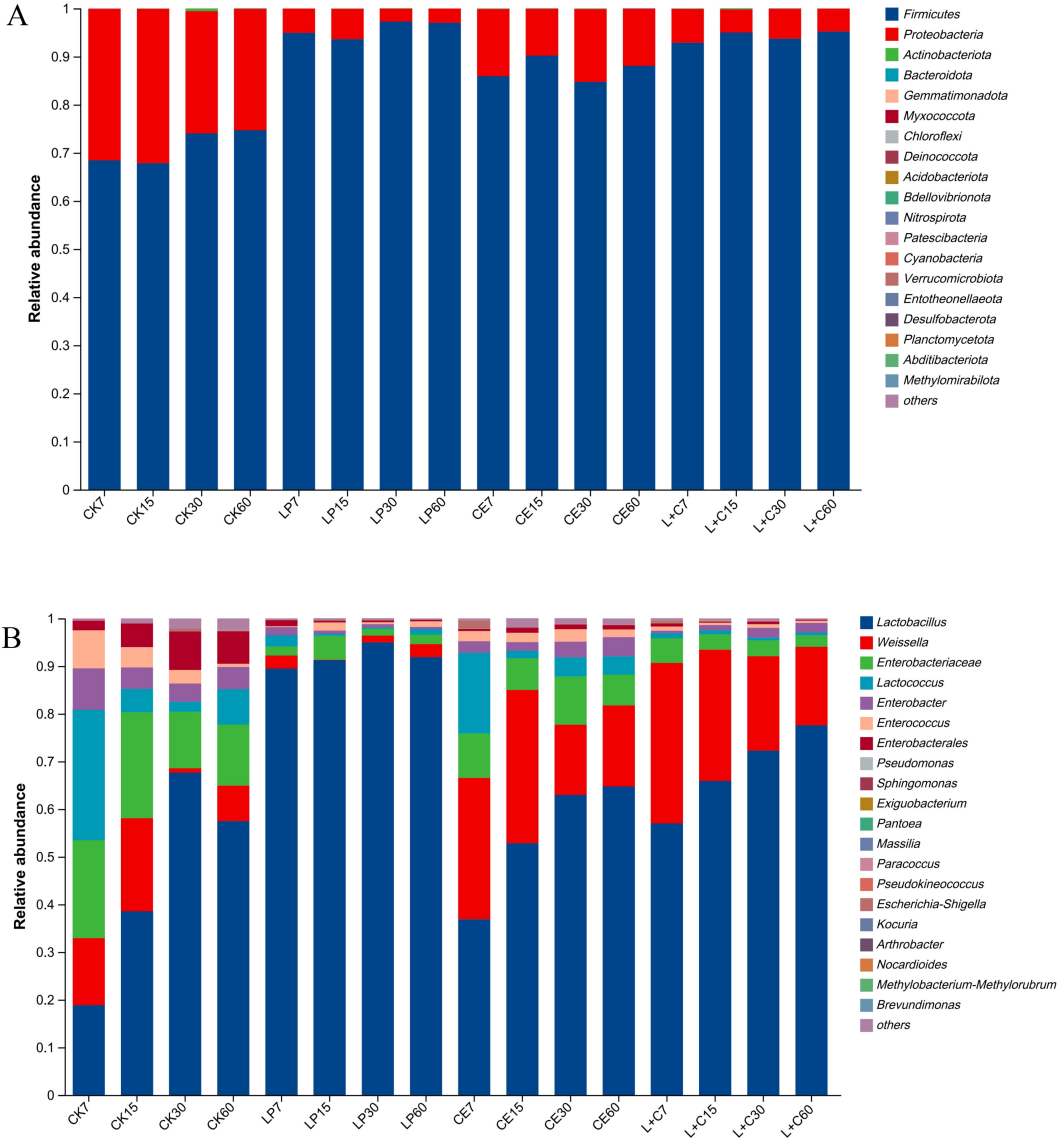
The bacterial abundance at phylum **(A)** and genus **(B)** composition of mixed silage. CK, no additive control; LP, *Lactobacillus plantarum*; CE, cellulase; L+C, *Lactobacillus plantarum* + cellulase.

The composition of the bacterial phylum levels is shown in **Figure 2A**. The dominant phylum in all groups were *Firmicutes* and *Proteobacteria*, and the abundance of *Firmicutes* in additive group was higher than that in CK group, and *Proteobacteria* in additive group was lower than that in CK group. The abundance of *Firmicutes* in LP group was higher than that in other groups, and that in CE group was lower than that in LP group and L+C group. The abundance of *Firmicutes* increased and *Proteobacteria* decreased during silage. In the CK group, *Firmicutes* were more abundant at days 30 and 60 days than at 7 days and 15 days. In the LP group, *Firmicutes* abundance was lower at 15 days of silage than at 7 days, 30 days, and 60 days. In the CE group, *Firmicutes* were more abundant at 15 days of silage than at 7 days, 30 days, and 60 days. In the L+C group, *Firmicutes* abundance was lower at 7 days silage than at 15 days, 30 days and 60 days.

The composition of the bacterial genus levels is shown in **Figure 2B**. The dominant genera in each group were *Lactobacillus*, *Weissella*, *Enterobacteriaceae*, *Lactococcus*, and *Enterobacter*. The *Lactobacillus* abundance in the additive group was higher than that in the CK group and the *Lactobacillus* abundance in the LP group was higher than that in the other groups. The abundance of *Enterobacteriaceae* and *Lactococcus* in group CK was high, and the abundance of *Weissella* in group CE and group L+C was high. In group CK, the *Lactobacillus* abundance at 7 days was low and reached the highest at 30 days, while the abundance of *Weissella*, *Enterobacteriaceae*, *Lactococcus*, and *Enterobacter* was high. In the LP group, the *Lactobacillus* abundance was consistently higher than that of other genera during silage. In CE group and L+C group, the abundance of *Lactobacillus* continued to rise during silage, while the abundance of *Weissella* and *Lactococcus* decreased.

### Functional prediction of bacterial communities of SA silage

**Figure 3:**
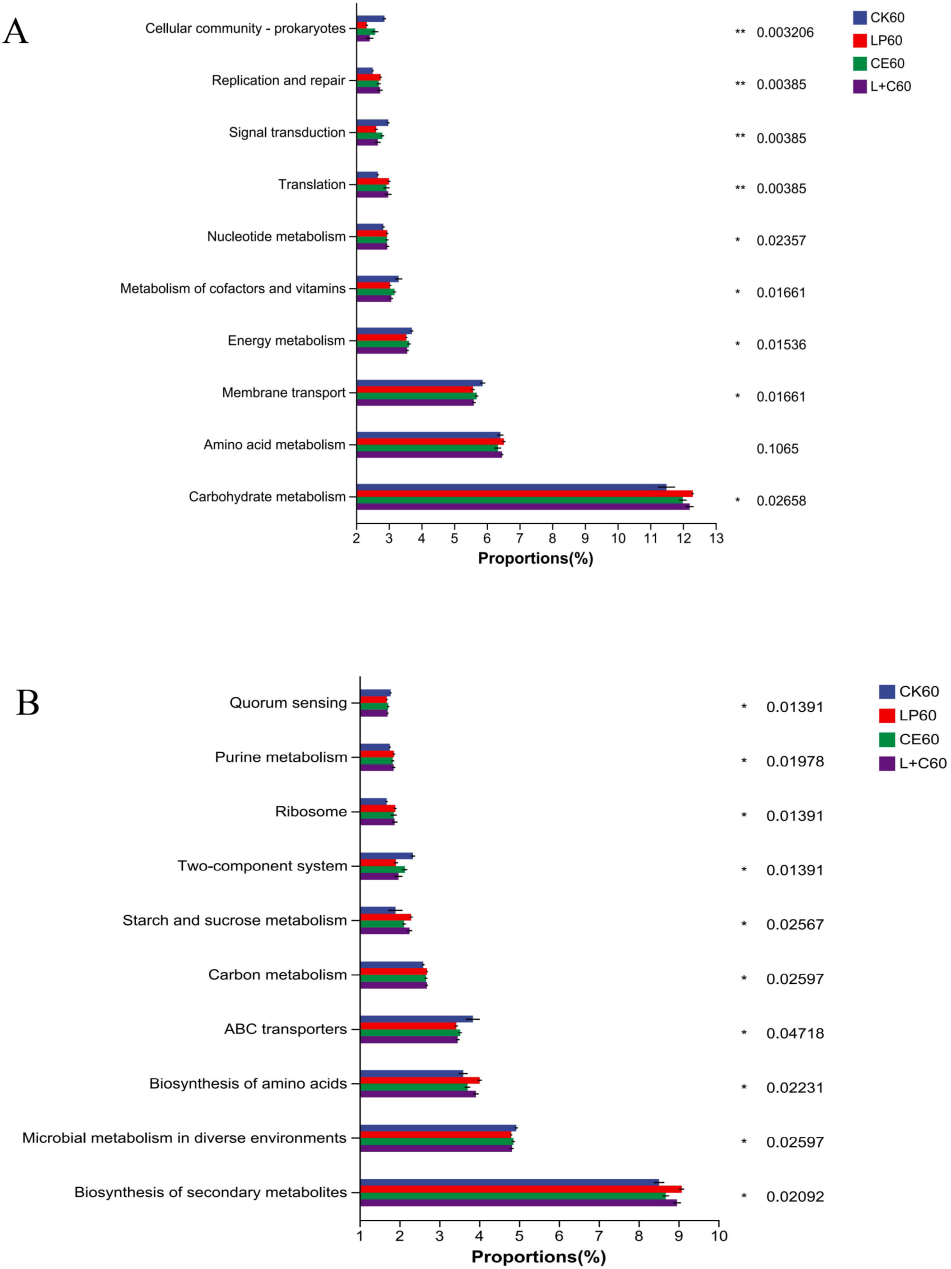
Functional prediction of bacteria at level 2 **(A)** and at level 3 **(B)**. CK, no additive control; LP, *Lactobacillus plantarum*; CE, cellulase; L+C, *Lactobacillus plantarum* + cellulase. *, significant at 0.05; **, significant at 0.01.

The results based on KEGG database at level 2 are shown in **Figure 3A**. At 60 days of silage, the main functions of each group were: carbohydrate metabolism, amino acid metabolism, membrane transport, energy metabolism, and metabolism of cofactors and vitamins. The carbohydrate metabolism of additive group was significantly higher than CK group, while membrane transport, energy metabolism, and metabolism of cofactors and vitamins was significantly lower than CK group. The carbohydrate metabolism of LP group and L+C group was higher, while membrane transport, energy metabolism, and metabolism of cofactors and vitamins were low. The results based on KEGG database at level 3 are shown in **Figure 3B**. At 60 days of silage, the main functions of each group were: biosynthesis of secondary metabolites, microbial metabolism in diverse environments, biosynthesis of amino acids, abc transporters and carbon metabolism. The biosynthesis of secondary metabolites, biosynthesis of amino acids, and carbon metabolism of additive group were significantly higher than CK group, while microbial metabolism in diverse environments and abc transporters were significantly lower than CK group. The biosynthesis of secondary metabolites and carbon metabolism of LP group and L+C group were higher, while microbial metabolism in diverse environments and abc transporters of cofactors and vitamins were low.

### Correlation between silage quality and bacteria at genus level

**Figure 4:**
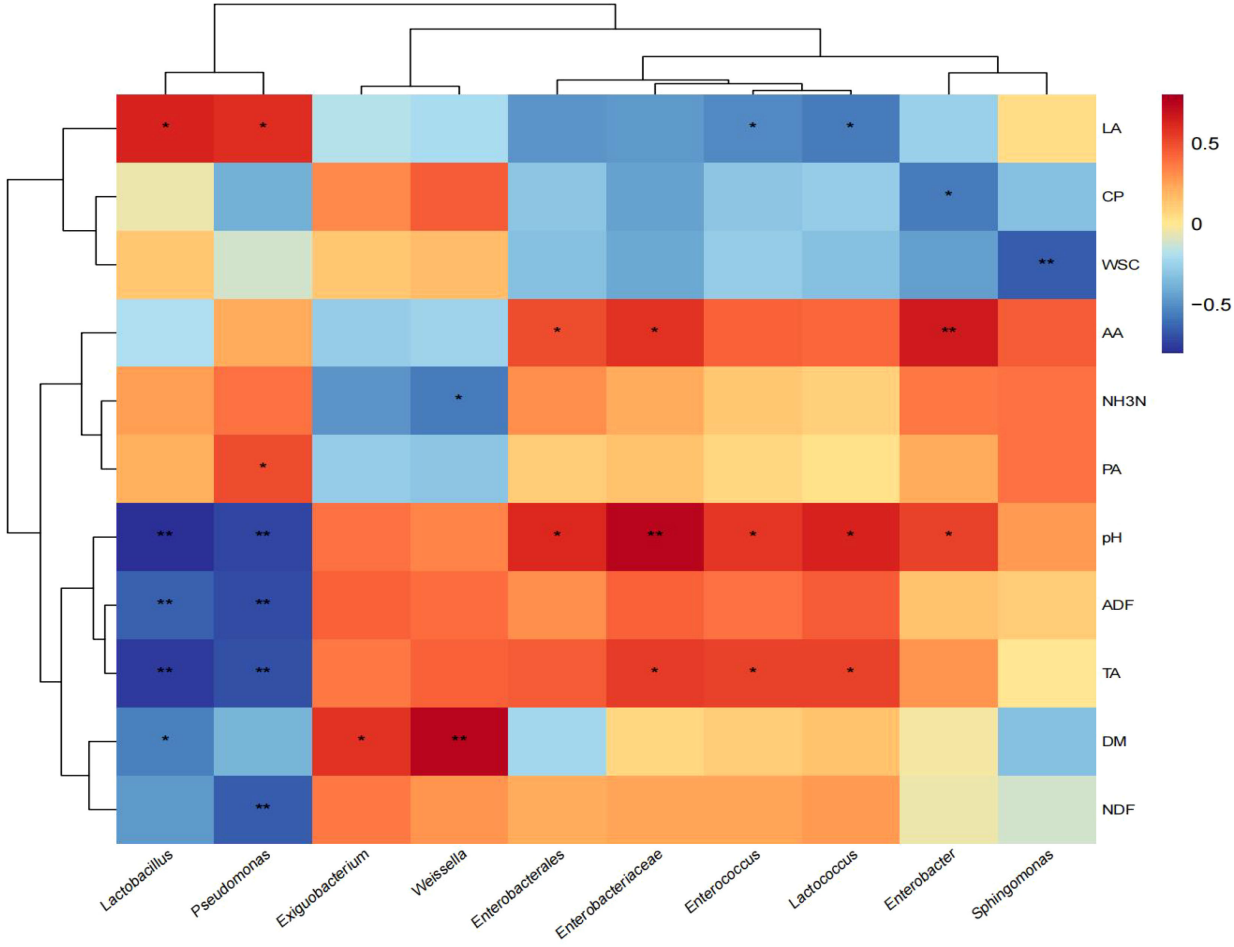
Correlation between silage quality and bacteria at genus level. DM, dry matter; CP, crude protein; NDF, neutral detergent fiber; ADF, acid detergent fiber; WSC, water-soluble carbohydrates; TA, total alkaloids; LA, lactic acid; AA, acetic acid; PA, propionic acid; BA, butyric acid; NH_3_-N, ammonia nitrogen. *, significant at 0.05; **, significant at 0.01.

The correlations between silage quality and bacterial genus are shown in **Figure 4**. *Lactobacillus* was positively correlated with LA (*p* < 0.05), while negatively correlated with DM (*p* < 0.05) and extremely negatively correlated with pH, ADF, and TA (*p* < 0.01). *Pseudomonas* was positively correlated with LA and PA (*p* < 0.05), and extremely significant negative correlation with pH, ADF, TA, and NDF (*p* < 0.01). *Weissella* was extremely positively correlated with DM (*p* < 0.01) and negatively correlated with NH_3_-N (*p* < 0.05). *Enterobacterales* and *Enterobacter* were significantly positively correlated with pH and AA (*p* < 0.05). *Enterobacteriaceae*, *Enterococcus*, and *Lactococcus* were significantly positively correlated with pH and TA (*p* < 0.05). *Sphingomonas* had a significant negative correlation with WSC (*p* < 0.05).

## Discussion

The examination of raw materials indicated that *S. rostratum* had elevated levels of soluble carbohydrates, neutral detergent fiber, acid detergent fiber, and total alkaloids, whereas alfalfa demonstrated larger concentrations of dry matter and crude protein. Soluble carbohydrates serve as the primary energy source for lactic acid bacteria during silage fermentation; inadequate levels may result in incomplete fermentation and insufficient acidity, thereby heightening the risk of spoilage and mold (Mahanna and Chase, 2003). Neutral detergent fibers and acid detergent fibers mostly comprise cellulose, hemicellulose, and lignin. Silage with elevated fiber content is less appealing, adversely impacting animal consumption and digestibility (Grant and Ferraretto, 2018). Numerous alkaloids possess bitter or otherwise unpalatable flavors, potentially diminishing the acceptability of silage. Moreover, specific alkaloids are poisonous and can induce animal poisoning, manifest central nervous system symptoms, and perhaps result in fatality in extreme instances (Cheeke, 1995). At the same time, the microbial count showed that the number of lactic acid bacteria was higher in *S. rostratum* and lower in alfalfa. More lactic acid bacteria in raw materials can help to accelerate the fermentation process, rapidly reduce pH value, and improve the storage quality and palatability of silage (You et al., 2022). The mixture of alfalfa and *S. rostratum* as silage raw materials can effectively reduce the content of fiber and alkaloid, increase the content of dry matter, crude protein and soluble sugar, improve the quality of raw materials, and promote the fermentation process of lactic acid bacteria.

The chemical composition analysis of silage indicated a declining trend in the levels of dry matter, crude protein, fiber, and soluble sugar throughout the silage process. From the commencement of the 15-day silage period, the chemical quality of the additive group consistently surpassed that of the control group, demonstrating superior nutrient retention. The inclusion of lactic acid bacteria as an additive accelerates the silage fermentation process, suppresses the growth and metabolism of detrimental microorganisms, hence enhancing the preservation quality of feed, corroborating the findings of Wang et al. (2022b) in whole corn silage. The addition of cellulase can break down complex polysaccharides into simple sugars by breaking down cellulose in plant cell walls, increasing the substrate available for fermentation (Li et al., 2018). In the additive group, the concurrent application of both agents enhances nutrient quality retention, as cellulase liberates additional sugars accessible to lactic acid bacteria by degrading fiber structures. This process accelerates the proliferation of lactic acid bacteria and the production of lactic acid, thereby more effectively preserving feed nutrition and mitigating material loss and spoilage risks during fermentation, corroborating the findings Xu et al. (2022) ‘s study on oat silage.

The analysis of fermentation components of silage showed that the pH of silage decreased, while lactic acid, acetic acid, propionic acid and ammonium nitrogen increased. The fermentation quality of additive group was always better than that of control group, and the fermentation effect was better. This is due to the fact that lactic acid bacteria produce a large amount of lactic acid through lactic acid fermentation, reducing the acidity of the silage environment (Okoye et al., 2023). The addition of cellulase can degrade a variety of sugars produced by fiber, and by participating in the synthesis of organic acids such as acetic acid and propionic acid, it can effectively inhibit the growth and metabolism of harmful microorganisms and avoid the occurrence of bad fermentation, which is consistent with the research results of Bai et al. (2023) in *Caragana korshinskii* silage. In the additive group, the fermentation effect of both is better, because cellulase can quickly form an anaerobic acidic environment by decomposing plant cell wall and promoting lactic acid fermentation, and the proliferation of lactic acid bacteria can inhibit the activities of harmful bacteria.

Alkaloids are secondary metabolites produced by plants, and high concentration of alkaloids may cause harm to the nervous system, digestive system and cardiovascular system of livestock (Poutaraud et al., 2017). The examination of alkaloid levels in silage revealed that as the duration of silage increased, the alkaloid content exhibited a declining pattern, with the additive group consistently demonstrating lower alkaloid levels than the control group. This occurs because certain alkaloids can be destroyed or altered by microorganisms, such as lactic acid bacteria, during fermentation, resulting in the formation of non-toxic or less toxic metabolites (Tao et al., 2020). Han et al. (2024) found similar results in the fermentation process of tea. At the same time, many alkaloids are unstable under acidic conditions and may undergo chemical decomposition or structural changes, resulting in reduced activity and toxicity (Basiliere and Kerrigan, 2020). The addition enhances the metabolic activity of lactic acid bacteria during silage fermentation and expedites the biodegradation and acidolysis of alkaloids. The alkaloid concentration in the *Lactobacillus plantarum* group was consistently lower than in the other groups, attributable to the rapid establishment of an acidic environment from the efficient fermentation by *Lactobacillus plantarum*, which facilitated alkaloid degradation. The sustained low pH and anaerobic conditions further inhibited the accumulation and regeneration of alkaloids, corroborating the findings of Klevenhusen et al. (2022) in the mixed silage of common ragwort (*Senecio jacobaea*) and eastern groundsel (*Senecio vernalis*), inoculum lactobacillus in silage promotes the degradation of alkaloids.

The results of silage α diversity showed that the community diversity of LP group was lower than that of other groups, and the community uniformity was higher than that of other groups. This is because *Lactobacillus plantarum* usually has strong competitiveness in the fermentation environment and can quickly dominate, thus inhibiting the growth of other microorganisms. This dominance can lead to a decrease in microbial diversity, as other types of microbes struggle to compete with *Lactobacillus plantarum* for resources (Xu et al., 2019). *Lactobacillus plantarum* concurrently generates metabolites, including lactic acid, via fermentation metabolism, thereby lowering the environmental pH and suppressing the proliferation of other acid-sensitive microorganisms, resulting in diminished diversity, corroborating the findings of Wu et al. (2022) in whole mulberry silage. Because the remaining microorganisms adapt to this acidic environment, the internal population structure of the dominant species is more stable and the uniformity is relatively high. The community diversity of the CE group was higher than that of the other groups, because the basal cellulase is an enzyme capable of breaking down cellulose, which is a complex carbon source that is difficult for many microorganisms to use directly. By adding cellulase, complex cellulose is broken down into simpler sugars, thus providing a more abundant and available nutrient source for a variety of microorganisms (Zhang et al., 2023). As cellulose decomposes, the variety of nutrient sources in the environment expands, allowing various microorganisms to inhabit distinct ecological niches. Certain microorganisms utilize the intermediate products of cellulose degradation, whereas others are better suited to the end products, this diversification of ecological niches enhances community diversity (Kukkar et al., 2022).

The analysis results of phylum level showed that abundance of *Firmicutes* in additive group was higher than that in control group and *Proteobacteria* was lower than that in control group. Additives can change the nutrient environment in silage, especially the effects on carbon and nitrogen sources. Many members of *Firmicutes*, especially lactic acid bacteria and other anaerobic fermenters, grow well in anaerobic environments and at lower pH levels, excelling at utilizing easily fermentable sugars and other simple carbon sources, which allows them to exhibit higher abundance in the additive group (Peng et al., 2024). Numerous bacteria within the *Proteobacteria* phylum necessitate intricate or particular nutritional requirements, and several species exhibit heightened sensitivity to oxygen or favor neutral over acidic environments (Li et al., 2021). Therefore, when additives alter the environment of silage, such as by promoting lactic acid fermentation and lowering pH, it may be detrimental to the growth of *Proteobacteria*, resulting in a lower abundance than the control group, which is the same pattern found in maize silage by Guo et al. (2022). The examination of genus-level composition indicated that the abundance of *Lactobacillus* in the LP group surpassed that of the other groups. The prevalence of *Enterobacteriaceae* and *Lactococcus* was greater in the CK group, while *Weissella* was more abundant in the CE and L+C groups. *Lactobacillus plantarum* can adapt to anaerobic growth conditions during silage, potentially providing it with a competitive edge over other strains, enabling rapid proliferation and increased abundance during silage (Keshri et al., 2018). At the beginning of silage, *Enterobacteriaceae* and *Lactococcus* gained a certain initial advantage, possibly due to a slower decline in acidity in the control group, resulting in a higher abundance, which is consistent with the changes of microbial community after adding *Lactobacillus plantarum* and *Lactobacillus buchneri* to silage by Drouin et al. (2019). *Weissella* may have a better ability to utilize cellulose degradation products as a source of nutrients for growth, thereby increasing its abundance in this group (Liao et al., 2022). The use of *Lactobacillus plantarum* and cellulase can effectively improve the composition of silage microbial community, inhibit the growth and metabolism of hybrid bacteria, and thus improve the effect of silage fermentation.

The microbial function study results indicated that glucose metabolism was elevated, but membrane transport, energy metabolism, and cofactor and vitamin metabolism were diminished in the additive group compared to the control group. Cellulase may enhance the availability of carbohydrates, including glucose and fructose, by degrading cellulose, thereby serving as energy and carbon sources for microorganisms like Lactobacillus plantarum, which promotes carbohydrate metabolism activity within the microbial community (Du et al., 2023). The low membrane transport may be due to the fact that the additives affect the absorption and excretion process of nutrients and metabolites by microorganisms, leading to the reduction of membrane transport function (Lei et al., 2024). The lower energy metabolism may be due to additives that change the way microorganisms use energy, such as reducing the activity of certain metabolic pathways, or inhibiting the activity of key enzymes related to energy metabolism, resulting in reduced energy metabolism of the microbial community (Wang et al., 2022a). The diminished metabolism of cofactors and vitamins may stem from additions that decrease microbial demand for specific cofactors and vitamins, consequently lowering metabolic activity towards these compounds (Labuschagne and Divol, 2021). Compared with the control group, the biosynthesis of secondary metabolites, amino acid biosynthesis and carbon metabolism were higher in the additive group, while the microbial metabolism and abc transporters in different environments were lower. This is because secondary metabolites are compounds synthesized by microorganisms under special environmental conditions and have a variety of biological activities and functions. *Lactobacillus plantarum* may promote the activity of microbial synthesis of secondary metabolites, thereby increasing the metabolic activity of this functional class (Raman et al., 2022). Amino acids serve as the fundamental components of proteins, and additives may have supplied the necessary ingredients and circumstances for their synthesis, hence facilitating the manufacture of amino acids within microbial communities, aligning with the findings of Zhao et al. (2022) ‘s study in amaranth silage. Carbon metabolism is one of the basic metabolic processes in the microbial community, and the degradation of cellulase may provide organic substances required for carbon metabolism in the microbial community, thus increasing the activity of carbon metabolism (Si et al., 2023). Microbial metabolism regulation in different environments is to adapt to specific environmental conditions and achieve optimal growth and survival. Additives change the adaptability and metabolic activity of microorganisms to different environmental factors, resulting in reduced microbial metabolism in a specific environment (Wu et al., 2020). ABC transporters are a category of membrane proteins prevalent in microorganisms, responsible for the transport of protons and ions. The additions may have modified the microbial community’s requirement for and transport of protons and ions, leading to diminished activity of ABC transporters (Keshri et al., 2019).

The results of correlation between silage quality and bacteria genus showed that *Lactobacillus* was positively correlated with LA and negatively correlated with DM, pH, ADF and TA. *Lactobacillus* is an important microorganism in silage fermentation, which produces lactic acid by fermentation of sugars during silage. The production of lactic acid can reduce the pH in the silage, inhibit the growth of other bacteria and molds, and help to maintain the quality and shelf life of the silage raw material (Muck et al., 2018). *Lactobacillus* may produce various degrading enzymes, promote the decomposition of cellulose and alkaloids in silage raw materials, and improve feeding value, which is consistent with the results obtained by Tao et al. (2020) in mixed silage of *Oxytropis glabra* and corn. *Pseudomonas* was positively correlated with LA and PA, but negatively correlated with pH, ADF, TA, and NDF. *Pseudomonas* had a positive correlation with LA and PA, while demonstrating a negative correlation with pH, ADF, TA, and NDF. *Pseudomonas* possesses a significant metabolic capacity, enabling it to breakdown complex organic compounds in silage raw materials, such as cellulose and other polysaccharides, hence enhancing the nutritional content of silage materials (Du et al., 2021). *Weissella* was positively correlated with DM and negatively correlated with NH_3_N. *Weissella* is abundant in the early stage of fermentation and can produce lactic acid and acetic acid through the fermentation process, and helps to reduce the pH value in the silage raw material, and gradually decreases with the extension of silage time, which is consistent with the results found by Mu et al. (2022) in mixed alfalfa and straw silage. *Enterobacter* and *Enterococcus* exhibited a positive association with pH, AA, and TA, and their competitive interactions with other bacteria during silage may influence the acidity and alkaloid content, indicating a favorable correlation. S*phingomonas* exhibits a negative correlation with WSC, as it decomposes proteins and carbohydrates in silage by the secretion of exogenous enzymes and proteases, resulting in silage spoiling. Certain *Sphingomonas* bacteria can digest sugars in silage, generating gases like carbon dioxide and methane, which leads to a reduction in the soluble carbohydrate content of silage (Liu et al., 2022). The analysis of the relationship between microorganisms and mixed silage components can provide a new idea for reducing alkaloid content and improving feed quality.

## Conclusion

During the fermentation process of the mixed silage of alfalfa and *S. rostratum*, *Lactobacillus plantarum* and cellulase can better preserve the contents of DM, CP and WSC, increase the content of LA, lower pH and the content of alkaloids, and enhance the fermentation quality. The application of additives augmented the abundance of *Lactobacillus* and *Weissella*, which are associated with the improvement of quality and the degradation of alkaloids. The differential microbial functions were mainly carbohydrate metabolism, biosynthesis of secondary metabolites and carbon metabolism. By combining nutritional quality, fermentation quality, total alkaloid content and microbial community analyses, it was determined that the addition of *Lactobacillus plantarum* alone or mixed addition of *Lactobacillus plantarum* and cellulase could obtain high-quality in mixed silage of alfalfa and *S. rostratum*.

## Data Availability

The original contributions presented in the study are included in the article/**Supplementary Material**. Further inquiries can be directed to the corresponding authors.
