# Supplementary material for: Influence of Lactobacillus plantarum and cellulase on fermentation quality and microbial community in mixed silage of Solanum rostratum and alfalfa
Source: Front Microbiomes. 2025 Jan 8;3:1510774. doi: 10.3389/frmbi.2024.1510774 (PMC12993579; doi:10.3389/frmbi.2024.1510774)

**Supplement data**

Influence of Lactobacillus plantarum and Cellulase on fermentation quality and microbial community in Mixed Silage of *Solanum rostratum* and alfalfa

Yuyu Li^1, 2^, Hua Wang^1, 2^, Yandong Zhang^1^, Yu Ji^1, 2^, Lizhu Guo^1, 2^, Lifen Hao^1, 2,*^ and Kejian Lin^1, 2,*^

*^1^ Institute of Grassland Research, Chinese Academy of Agricultural Science, Hohhot 010010, China*

*^2^ Key Laboratory of Biohazard Monitoring, Green Prevention and Control for Artificial Grassland, Ministry of Agriculture and Rural Affairs, Hohhot 010010, China*

*Correspondence: linkejian@caas.cn (K.L.); haolifen@caas.cn (L.H.)

**Figure. S1.** Normality test of chemical composition and fermentation composition data of mixed silage of *Solanum rostratum* and alfalfa. DM, dry matter; CP, crude protein; NDF, neutral detergent fiber; ADF, acid detergent fiber; WSC, water-soluble carbohydrates; LA, lactic acid; AA, acetic acid; PA, propionic acid; NH_3_-N, ammonia nitrogen.


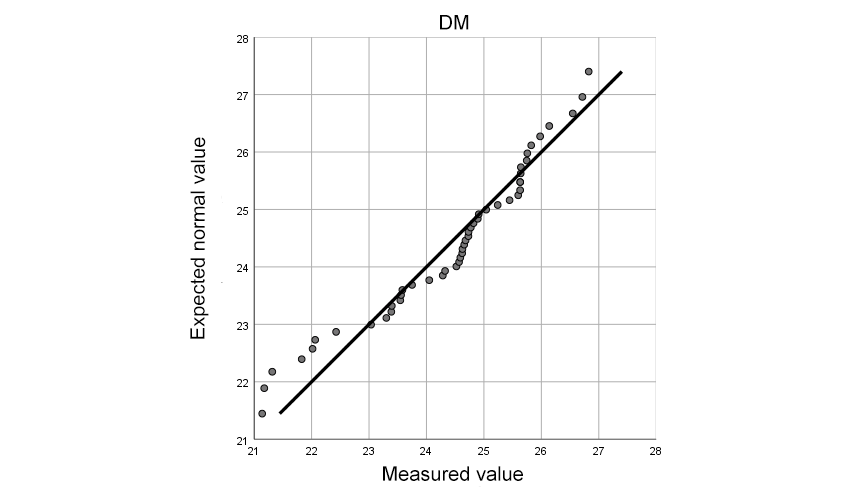

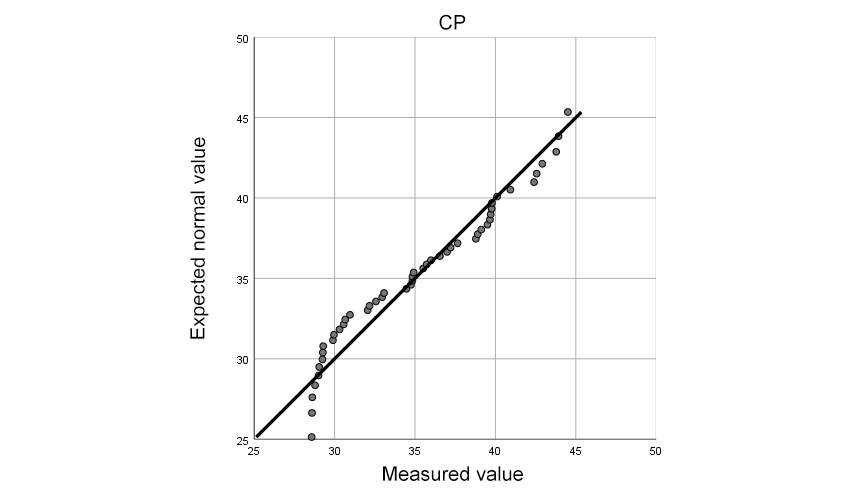

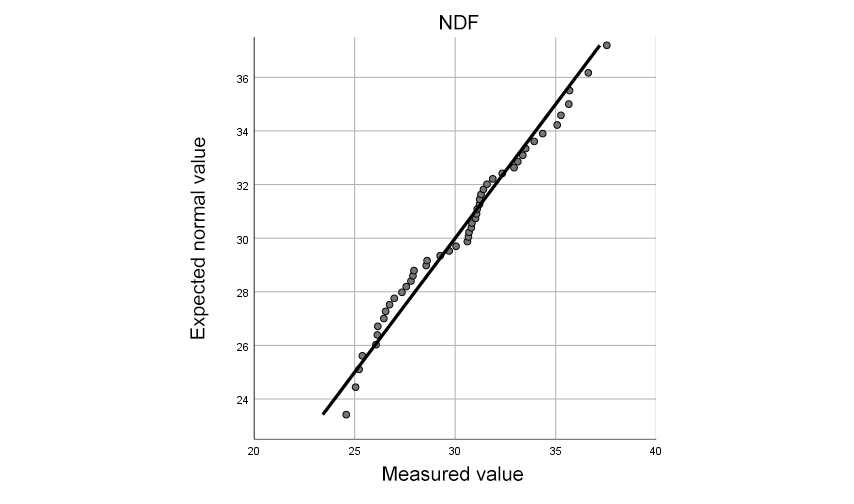

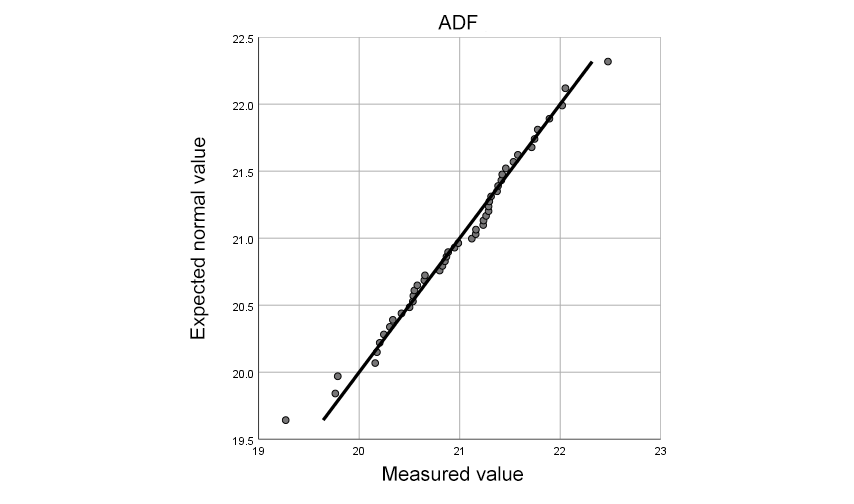

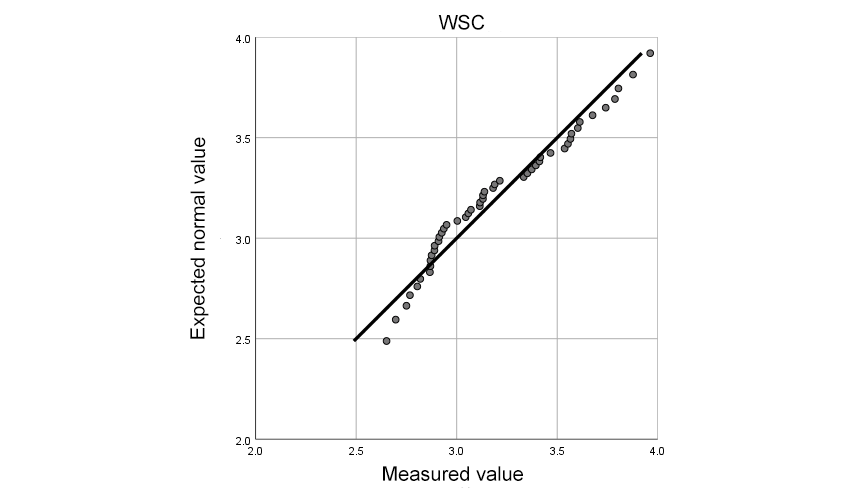

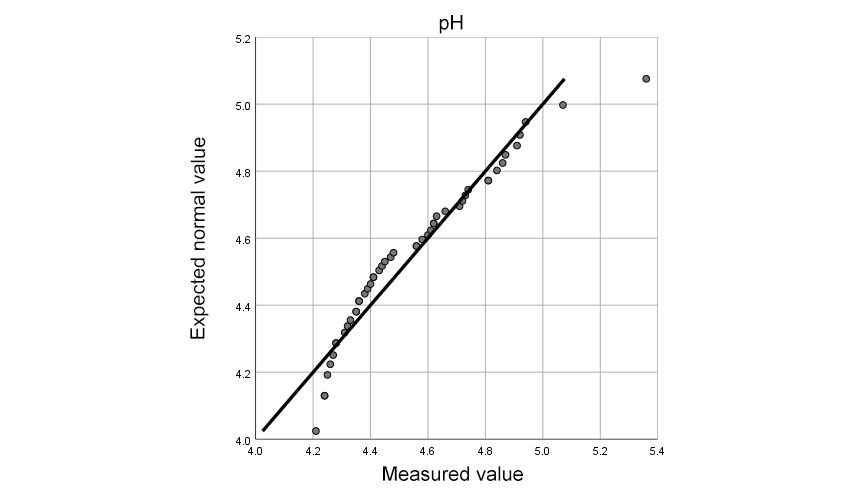

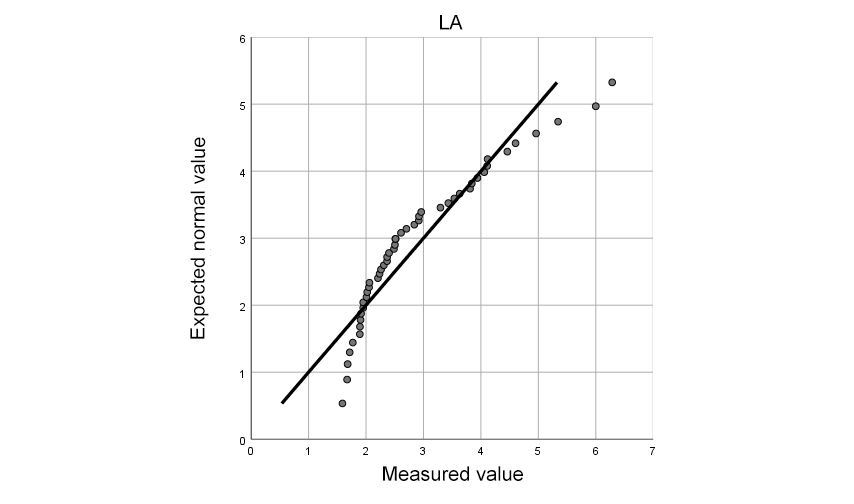

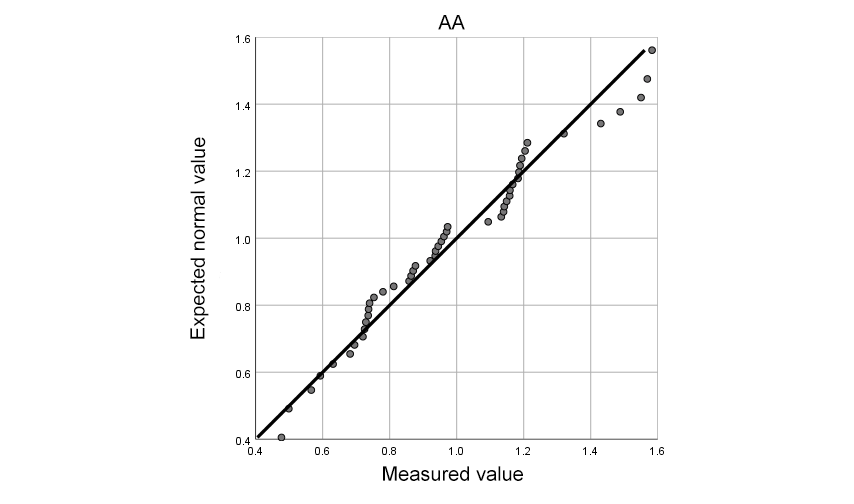

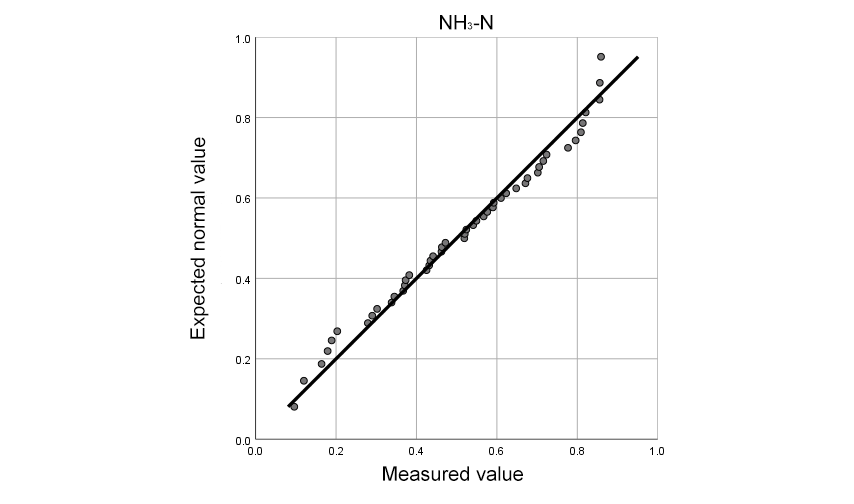

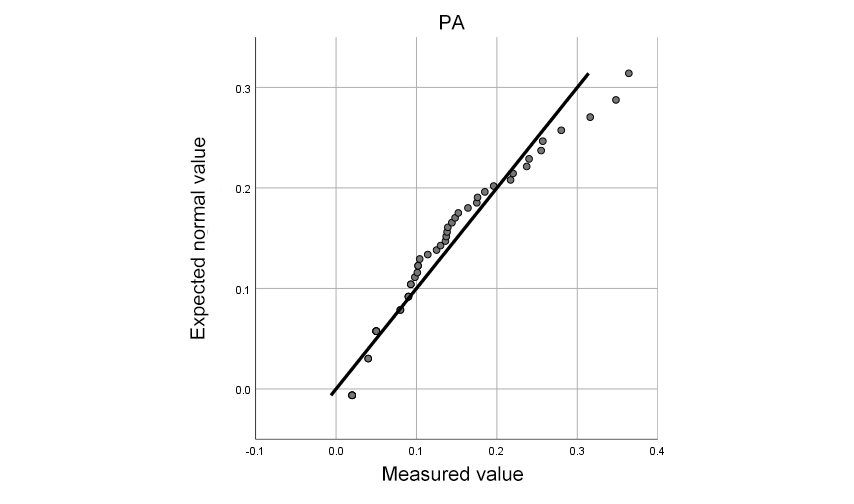

Supplement: Supplementary file 1 [file DataSheet1.doc]
